# Graph-Theoretical Study of Functional Changes Associated with the Iowa Gambling Task

**DOI:** 10.3389/fnhum.2016.00314

**Published:** 2016-06-27

**Authors:** Taylor Bolt, Paul J. Laurienti, Robert Lyday, Ashley Morgan, Dale Dagenbach

**Affiliations:** ^1^Department of Psychology, University of MiamiCoral Gables, FL, USA; ^2^Department of Radiology, Wake Forest School of MedicineWinston-Salem, NC, USA; ^3^Department of Psychology, Wake Forest UniversityWinston-Salem, NC, USA

**Keywords:** graph theory, Iowa Gambling Task, resting state fMRI, fronto-parietal network, decision-making

## Abstract

The primary aim of this study was to examine changes in functional brain network organization from rest to the Iowa Gambling Task (IGT) using a graph-theoretical approach. Although many functional neuroimaging studies have examined task-based activations in complex-decision making tasks, changes in functional network organization during this task remain unexplored. This study used a repeated-measures approach to examine changes in functional network organization across multiple sessions of resting-state and IGT scans. The results revealed that global network organization shifted from a local, clustered organization at rest to a more global, integrated organization during the IGT. In addition, network organization was stable across sessions of rest and the IGT. Regional analyses of the Default Mode Network (DMN) and Fronto-Parietal Network (FPN) revealed differential patterns of change in regional network organization from rest to the IGT. The results of this study reveal that global and regional network organization is significantly modulated across states and fairly stable over time, and that network changes in the FPN are particularly important in the decision-making processes necessary for successful IGT performance.

## Introduction

The Iowa Gambling Task (IGT) is a popular paradigm used to assess real world decision-making by clinicians and researchers (Bechara et al., 1994). In this task, individuals are instructed to consecutively select cards from four decks (typically referred to as A, B, C, and D). Each deck is associated with varying degrees of cash rewards and punishments after each card selection. Decks A and B yield large cash rewards, but large penalties, resulting in a net loss; decks C and D yield small cash rewards, but small penalties, resulting in a net gain. Successful decision making is measured by the difference between the number of selections from “advantageous” decks (C and D) and the number of selections from “disadvantageous” decks (A and B; Bechara et al., 1994). A variety of methods have been used to identify the relevant brain areas associated with IGT performance. Early research on the neural substrates of the IGT used primarily lesion studies (Bechara et al., 1994, 1998, 1999; Manes et al., 2002). Later studies have used functional neuroimaging techniques, particularly PET and fMRI, as a convergent methodology that allows for more precise measurements of the brain areas contributing to the task, and without the disadvantages associated with lesion studies (Rogers et al., 1999; Bolla et al., 2003; Windmann et al., 2006). The present study applies the more recent technique of network analysis to explore functional network organization among the brain areas that support performance on the IGT (Bullmore and Sporns, 2009; Guye et al., 2010).

Graph-theoretical study of functional network organization has been primarily applied to resting-state fMRI, but recently has been extended to task-associated changes in network organization (Bassett et al., 2011; Stevens et al., 2012; Cole et al., 2013; Rzucidlo et al., 2013; Stanley et al., 2014). The present study extends the graph-theoretical study of task-related changes in network organization to the examination of decision-making under uncertainty using the IGT. While there has been extensive research on the neural activations associated with decision-making behavior in the IGT, much less is known about the functional integration that occurs between those brain areas implicated in activation studies. The goal of this study was to explore changes in the extent and nature of the functional integration that occurs in the brain between rest and the IGT.

Networks analyses of functional brain networks are typically applied globally, meaning networks are described by metrics (*K, E_glob_, E_loc_*, etc.) averaged across the entire network. At the global level (i.e., whole-brain network), several studies have found that there are functionally important changes in network organization from rest to task-states (Rzucidlo et al., 2013; Stanley et al., 2014, 2015; Wen et al., 2015). However, global metrics may overlook significant regional changes in the network (Moussa et al., 2011; Rzucidlo et al., 2013). Of additional interest are changes in the spatial distribution of high degree nodes across rest and IGT states, as well as changes in the network structure of *a priori* defined networks of interest. Two major networks of interest are the Default Mode Network (DMN) and Fronto-Parietal Network (FPN). The DMN tends to be activated while participants are at rest and correspondingly deactivated during most task states, and is thought to correspond to internally-oriented mentation (Raichle et al., 2001; Buckner et al., 2008). The DMN is thought to consist of the vmPFC, inferior parietal cortex (IPC), and precuneus/posterior cingulate cortex (Raichle et al., 2001; Fox et al., 2005; Buckner et al., 2008). One such area, the vmPFC, is consistently implicated in the decision-making processes of the IGT (Bechara et al., 1994, 1999; Ernst et al., 2002; Lin et al., 2008; Li et al., 2010). In addition, the IPC, an area observed to be active during the IGT (Ernst et al., 2002; Lin et al., 2008), is also a crucial component of the DMN. How functional organization in the DMN is modulated from rest to task-states is a question still under active investigation. Sustained connectivity between areas of the DMN persists from rest to most task states, including working memory, visual classification, and semantic decision making; and has been shown to correlate with behavioral outcomes (Fransson, 2006; Fransson and Marrelec, 2008; Harrison et al., 2008; Hasson et al., 2009; Gao et al., 2013; DeSalvo et al., 2014). A more recent study (Rzucidlo et al., 2013) examining functional network changes from rest to a 2-back working memory task, indicates that hub nodes in the DMN (i.e., the precuneus) may change from rest to task states. However, network analyses of DMN functional organization during other complex tasks, such as the IGT, are yet to be performed.

The FPN consists of the dorsolateral prefrontal cortex (DLPFC), superior parietal cortex (SPC), ventral lateral prefrontal cortex (VLPFC), and dorsal anterior cingulate cortex (dACC; Damoiseaux et al., 2006; Dodds et al., 2010; Power et al., 2011; Cole et al., 2013; Scolari et al., 2015). The FPN is linked to a variety of cognitive control functions, including attention, inhibition, working memory, and response control. The cognitive processes involved in the IGT, such as response control, judgments of probabilities, and the maintenance of previous reward outcomes in short-term memory, all require the operation of the FPN. The DLPFC, a crucial component of the FPN, is an area consistently implicated in the decision-making processes of the IGT (Ernst et al., 2002; Manes et al., 2002; Li et al., 2010). In addition, activation in the ACC (primarily the dorsal ACC) has been consistently observed during the IGT (Ernst et al., 2002; Tanabe et al., 2007; Lin et al., 2008; Li et al., 2010) Little research has been conducted to examine the changes in functional network organization of the FPN across states. Larson-Prior et al. (2009) found that the executive control network (i.e., FPN) exhibited a decrease in correlation strength from wake to light sleep. On the other hand, Sala-Llonch et al. (2012) found that correlations within the FPN increased from rest to a 3-back working memory task. Studies of changes in functional network organization have found that the FPN may be highly adaptable to changes in task demands found that the FPN is highly adaptable to different task demands, shifting its functional organization across task states (Cole et al., 2013; Rzucidlo et al., 2013). However, network analysis of FPN functional organization during a complex decision-making task, with multiple cognitive control components, such as the IGT, is yet to be performed.

One recent study did use a network analysis approach to examine the consistency of communities of strongly correlated brain regions during two sessions of the IGT for both young and older adults (Moussa et al., 2011). Although not examining differences in community structure across rest and the IGT, they found that a net increase in connectivity in the vmPFC between two sessions of the IGT was positively correlated with a net positive change in the IGT performance between the two sessions. Thus, connectivity changes across IGT tasks seem to be functionally associated with performance in the task.

The present study extends the network analysis of the IGT to the examination of connectivity changes across rest and the IGT using a repeated-measures design with repeated observations of both rest and IGT states for each participant. The repeated-measures design of the study allows for the examination of change/stability in network organization between rest and task states, as well as the same state over several sessions. For the present study, whole brain metrics (*E*_loc_ and *E*_glob_) were used to examine change/stability in overall network organization across the entire brain. It was predicted that network organization would shift from a more local, modular organization at rest to a more global, integrated organization during the IGT. In addition, between and within task changes in network organization within areas of the DMN and FPN were further examined. Consistent with its central role in cognitive control during task states (Cole and Schneider, 2007), FPN was predicted to increase in functional significance, as measured by network metrics (*K, E_loc_*, *E*_glob_), from rest to IGT. In order to establish the functional significance of these network metrics for IGT performance, performance on the IGT was correlated with connectivity in areas of the DMN and FPN.

## Materials and Methods

### Participants

Participants were recruited from the Wake Forest University Introductory Psychology research participant pool and the community. Participants from the research participant pool received course credit, and community members of Winston-Salem, North Carolina were given monetary compensation in return for their participation. The study was approved by the Institutional Review Board of Wake Forest School of Medicine. Data was collected from nine young (7 Male, *M_age_* = 19.9, range: 19–23) healthy adults, not including one participant’s data that was excluded due to excessive motion. Participants were provided with a Safety Screening Form and Medical History Questionnaire to complete prior to the experiment. In addition, participants were screened for corrected visual acuity, small to moderate hearing loss, and right-handedness prior to the experiment. Participants were provided with an additional Safety Screening Form upon the day of their fMRI scan. Any participants who had implants, devices, or objects that would interfere with the fMRI procedure were excluded from the experiment.

### Imaging Design

#### Scanner Experiment

Prior to the fMRI scan, participants viewed a description of the experiment on a PowerPoint slideshow and participant questions regarding the experiment were answered. Participants were provided with fMRI compatible goggles, ear plugs, and a hand-held button response box. An anatomical brain scan was collected for each participant (5 min) at the beginning of the scan. Subsequently, participants alternated between a resting state during which they looked at a fixation cross without moving (4 min), and the IGT (4 min). The participants completed four resting blocks, four IGT blocks, and four spatial-orienting blocks (scans not included in the analysis), always in the same order: rest, IGT, spatial orienting. In addition, a perfusion sequence was collected at the end of the experimental design, during which participants rested quietly with their eyes closed for 5 min (this scan was not included in the analyses). The total length of time each participant was in the scanner was 58 min. Data from the spatial orienting task and the perfusion task were not included in the present set of analyses.

### Materials

The resting-state condition consisted of the participant laying quietly in the scanner and staring at a fixation cross. Participants were instructed to stay awake and let their thoughts wander during the duration of the scan. Because participants were in the fMRI scanner while they were performing the IGT task, a computerized version was used. In the computerized task, participants indicated their card selection (decks were represented on the monitor) by pressing one of four buttons on a response box that indicated their answers while in the scanner. For each trial, the participant was given 2 s to select a card, and after each response, a screen appeared displaying whether the response resulted in a win or loss, the win/loss amount, and their total earning. At the beginning of the task, participants started with $0 and were told that the game consists of a long series of card selections from four decks of cards (decks 1, 2, 3 and 4) displayed on the screen. After selection of each card by pressing the corresponding button on the button box, the participant received a certain amount of money that varies with each deck. Selection from decks 1 and 3 yielded a reward of $150, while selection from decks 2 and 4 yielded a reward of $200. After selecting some cards, the subjects may have incurred a penalty, and the penalty amount varied with each deck. Penalty amounts were higher in the high-paying decks (2 and 4), and lowest in the low-paying decks (1 and 3). Because of the higher penalty cost of the higher-paying decks, the long-term yield was a net gain for the low-paying decks, and a net loss for the high-paying decks. The decks also varied in the frequency of penalties. Decks 1 and 2 have less frequent penalties, while decks 3 and 4 had more frequent penalties. Decks 2 and 4 are referred to as “disadvantageous” because they incurred a net loss for the participant, and Decks 1 and 3 are referred to as “advantageous” because they incurred a net gain for the participant. Displays were presented and responses were recorded using Eprime 2.0 software.

### MR Image Acquisition

All imaging was performed on a Siemens SKYRA 3T MRI scanner using a GE eight channel neurovascular head coil. The protocol parameters for the anatomical scan were the following: phase/frequency = 256/256; 156 contiguous slices, 1.0 mm thick; in-plane resolution of 0.938 mm × 0.938 mm; echo time (TE) = 4.74 ms; repetition time (TR) = 4.68 ms; Inversion time (TI) = 600 ms. Whole-brain gradient echo echo-planar imaging (EPI) was used to detect blood-oxygen-level-dependence (BOLD) fMRI signal changes during each task. The EPI contained the following parameters: 120 volumes with 35 contiguous slices per volume; slice thickness 4.0 mm; in-plane resolution of 4 × 4 mm; TR/TE = 2000/25 ms. The voxels of each anatomical image were identified by using the Automatic Anatomical Labeling (AAL) atlas. All anatomical and functional image processing was done using SPM 8, Statistical Parametric Mapping (Friston et al., 1994). Further processing for network statistics were completed using MATLAB scripts.

### Functional Image Pre-processing

The first 10 volumes of the functional images were discarded prior to preprocessing. The remaining functional images were realigned to the first image volume using “rigid body” transforms to remove head motion. Next, the EPI image for each participant was co-registered to the structural image. The structural image was normalized to the standard stereotactic Montreal Neurological Institute (MNI) space, and warping parameters. The images were not smoothed to avoid spurious increases in local connectivity (Hayasaka and Laurienti, 2010). Functional volumes with excessive motion were removed according to procedures described in Power et al. (2012). The time courses were extracted for each gray matter voxel based on the AAL atlas and band-pass filtered to remove signals outside the range of 0.009–0.08 Hz (Tzourio-Mazoyer et al., 2002). White matter, cerebrospinal fluid and motion parameters were regressed from the time series to remove spurious signals associated with physiological noise such as heart beat and respirations and motion. In addition, regression of the global signal was performed because it has been shown to reduce bias in network. parameters (Hayasaka, 2013) and enhances signal-to-noise separation (Shirer et al., 2015).

### Network Construction and Analysis

Pre-processed functional data were masked such that only gray matter voxels were included. This was achieved by first summing the gray mater, white matter and cerebrospinal segment maps to generate a binary whole-brain mask. This mask was then intersected with gray matter areas specified by the AAL atlas. Then the white matter segment was subtracted (thresholded at 99%) to remove subject-specific white matter edges that may coincide with the AAL gray matter atlas.

The gray matter voxels were then used in the construction of the network for each participant. Networks are represented as graphs consisting of nodes and edges. Each voxel represents a node, and a network is constructed through a correlation matrix containing Pearson correlation coefficients between each voxel’s time series. The correlations matrices were thresholded using the following equation: *N* = *K^S^*, where *S* is the equivalent of the shortest path length in a random network, *N* = number of nodes, and *K* = degree. The correlation coefficient that satisfied *N* = *K^S^* was used as a lower bound when creating binary adjacency matrices (Hayasaka and Laurienti, 2010). This thresholding procedure ensures that the connection densities are consistent across conditions and individual subjects in the event that there is a change in the number of network nodes. For networks with the same number of nodes, this is the equivalent of fixing the average *K* across networks (Simpson et al., 2013a). Network properties were measured at all thresholds in order to determine whether those properties are independent of threshold effects. Those coefficients in the correlation matrix above the threshold received a 1 (i.e., representing an edge between those voxel pairs), and those coefficients below received a 0 (i.e., no connection). The threshold *S* = 2.5 (from the equation above) was used for this article. This threshold was chosen based on previous research showing that networks thresholded with an *S* greater than 3 tend to fragment (Hayasaka and Laurienti, 2010). A network was created for each block (Rest 1, IGT 1, Rest 2, IGT 2, etc.), resulting in 12 networks for each participant and 108 networks overall (*N* = 9).

### Creation of DMN and FPN Masks

**Figure 1:**
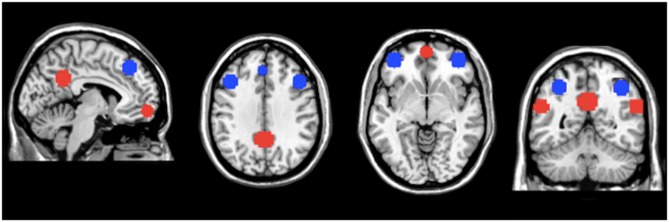
**Masks of the default mode network (DMN) and fronto-parietal network (FPN).** Regions of interest (ROIs) for the DMN (in red) and FPN (in blue) used for regional analyses. The network metric values within these regions of the DMN and FPN were averaged to generate mean values for the entire DMN and FPN, respectively.

Masks for areas of the FPN and DMN were created using WFU-pick atlas software (Maldjian et al., 2003; Figure 1). Ten millimeter spheres were placed in areas of the AAL atlas corresponding to important regions of the DMN and FPN (Fox et al., 2005; Buckner et al., 2008; Vincent et al., 2008; Yeo et al., 2011) For the FPN, the regions of interest (ROIs) were generated using a 10 mm sphere placed at ±40, 48, −5 for the right and left ventrolateral prefrontal cortex (lateral inferior frontal gyrus); ±43, 22, 34 for the right and left dorsolateral prefrontal cortex (lateral middle frontal gyrus); 37, −61, 48 for right SPC (dorsal posterior parietal cortex) and −32, −61, 48 for left SPC; −2, 35, 43 for the dACC/dorsomedial prefrontal cortex. For the DMN, the ROIs were generated using a 10 mm sphere placed at ±55, −57, 25 for the left and right IPC; 0, 57, −10 for the vmPFC; 0, −51, 31 for the precuneus/PCC. For FPN and DMN comparisons, all the individual ROI’s were merged within each network, resulting in a single mask for both the FPN and DMN. The networks masks were used to extract the network metric of interest from each voxel in the mask. The values were then averaged to generate a mean value for each network.

### DMN and FPN Correlations with IGT Performance

To establish the functional significance of connectivity metrics within the DMN and FPN, a linear mixed-model predicting IGT performance (i.e., advantageous minus disadvantageous decks) was conducted with subject modeled as a random effect, and the network metrics (*K, E_glob_, E_loc_)* from each network (DMN or FPN) modeled as simultaneous covariates (fixed-effects). In addition, to account for correlated errors associated with each individual a first order autoregressive or AR(1) covariance structure was specified to model correlations between previous time points.

### Network Metrics

Below is a brief definition of the network metrics calculated for each network (for detailed information on these and additional metrics see Bullmore and Sporns, 2009; Telesford et al., 2011):

Degree (*K*)—the number of edges (i.e., connections) of a node.

Global Efficiency (*E*_glob_): is the inverse of characteristic path length, which is a measure of the average number of minimum connections that should be passed to join any two nodes in a network (Latora and Marchiori, 2001). It is a scaled measure that ranges from 0 to 1, with a value of 1 signifying maximum distributed processing.

Local Efficiency (*E_loc_*): is the inverse of the characteristic path length connecting all neighbors of that node (Latora and Marchiori, 2001). It is a scaled measure ranging from 0 to 1, with a value of 1 signifying a node with solely local connections.

#### Degree Centrality Maps

To further explore differences in network organization between rest and task states, differences in the spatial distribution of hub nodes between rest and IGT networks were assessed. The “hubness” of a node was calculated using *degree centrality* (i.e., simply the number of connections for each node). Hubs for each network were identified as those nodes that were included in the top 20% degree distribution of that network. In order to test the differences between the spatial distribution of hub nodes between rest and task states a recently developed permutation testing framework for comparing differences in network organizations between groups was used (Simpson et al., 2013b). We briefly describe the approach here. The Jaccard index (JI) was used to quantify similarity of the spatial pattern of degree hubs. This comparisons was made between all networks across participants and tasks. This yielded a similarity matrix. Then the average similarity within task was divided by between task to generate a comparison statistic (analogous to an ANOVA). To determine if there was a difference between the tasks the permutation procedure was used. The task labels were permuted and the statistic was computed again for the permuted sample. This was repeated 512 times to build a null distribution. We then compared our true value to the null distribution to determine significance.

## Results

### Behavioral Performance

**Figure 2:**
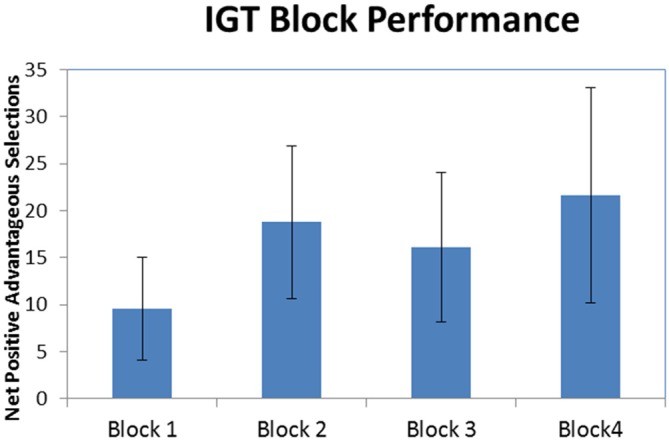
**Iowa Gambling Task (IGT) block performance.** Net-positive advantageous selections across all four blocks of the IGT (error bars represent standard error).

Figure 2 illustrates that, on average, participants made more selections from the good decks (1 and 3) compared to the bad decks (2 and 4) in all four blocks of the IGT (M_1_ ± SE = 9.56 ± 5.44, M_2_ ± SE = 18.78 ± 8.15, M_3_ ± SE = 16.11 ± 7.94, M_4_ ± SE = 21.67 ± 11.45). As can be seen in the standard error bars of the chart, variability in performance among the participants was high. A one-way repeated-measures ANOVA was conducted to test for differences in advantageous vs. disadvantageous card selections among the four blocks of the IGT. The results indicated there were no significant difference among the four blocks of the IGT (*F*_(3,24)_ = 1.094, *p* = 0.371). While there was no statistically significant learning effect, the prevalence of net-positive advantageous selections indicates participants were engaged in the task.

### Whole-Brain Network Metrics

For each participant, whole-brain network metrics were generated for mean *E_glob_* and *E*_loc_ for each IGT and rest session (differences in *K* were not examined due to the fact that *K* is controlled at the whole-brain level; see “Materials and Methods” Section). 2 (condition) × 4 (session) repeated measures ANOVAs (one for both *E*_loc_ and *E*_glob_) were used to determine whether any of these metrics varied significantly across the four rest and four IGT sessions, as well as whether they varied between the conditions.

Across sessions, none of the metrics exhibited significant differences (*E_loc_*: *F*_(3,24)_ = 1.154, *p* = 0.348, *η*^2^ = 0.126; *E*_glob_: *F*_(3,24)_ = 1.593, *p* = 0.217, *η*^2^ = 0.166). These results suggest that overall network organization across sessions from the same task (both rest and IGT) remained consistent.

There were significant main effects of task for both metrics. Consistent with our hypotheses, the results indicated that local efficiency (*E_loc_*) decreased from rest (M ± SE = 0.52 ± 0.005) to IGT (M ± SE = 0.502 ± 0.004) states, *F*_(1,8)_ = 9.12, *p* = 0.017, *η*^2^ = 0.533. However, although a decrease from rest to IGT was observed across thresholds, the significance of this decrease was not found to be independent of threshold effects, thus, these results should be interpreted with caution (*p* = 0.017, *p* = 0.063 and *p* = 0.122 for *S* = 250, 300, 350, respectively). Also consistent with our hypotheses, global efficiency (*E*_glob_) increased from rest (*M* ± *SE* = 0.189 ± 0.005) to IGT (*M* ± *SE* = 0.213 ± 0.009) *F*_(1,8)_ = 7.45, *p* = 0.026, *η*^2^ = 0.482. There was no significant interaction between task and session for either metric.

### Location of Central Nodes in Rest and IGT States

**Figure 3:**
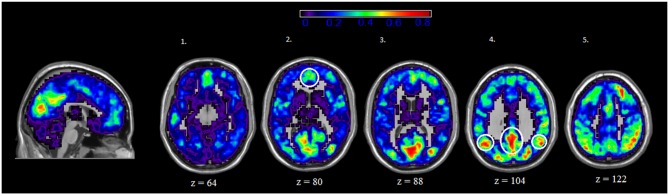
**Rest—Spatial Consistency of hub areas.** Average degree across all participants and sessions at rest, with areas of the DMN circled in yellow. Areas of the DMN, inferior parietal cortex (IPC; axial slice 4—lateral), Precuneus/PCC (axial slice 4—medial), and medial (ventral and dorsal) prefrontal cortex (axial slice 2) exhibit a high degree of connectivity. In addition, there is high degree of connectivity in the visual cortex.

**Figure 4:**
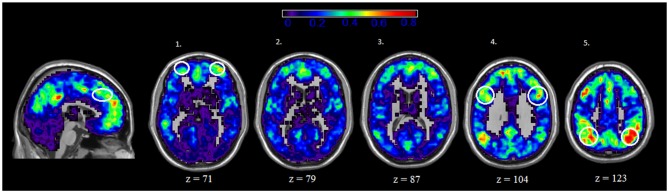
**IGT—Spatial Consistency of hub areas.** Average degree across all participants and sessions during IGT state, with areas of the FPN highlighted yellow. Examination of the both Rest and IGT consistency images reveal important shifts in the consistency of hub nodes. In particular, areas of the FPN, Dorsal anterior cingulate cortex (dACC; sagittal slice) ventral lateral prefrontal cortex (VLPFC; axial slice 1), dorsolateral prefrontal cortex (DLPFC; axial slice 4), and superior parietal cortex (SPC; axial slice 5) exhibit a higher degree of connectivity during IGT. Interestingly, the medial prefrontal cortex (ventral and dorsal) exhibits high consistency of hub nodes in both the IGT and rest.

We also examined the spatial distribution of hubs, or nodes with the highest degree (i.e., connections) in both rest and IGT states. For both rest and IGT conditions, the spatial consistency of the top 20% degree nodes across all rest and IGT networks were calculated. Figures 3, 4 display the consistency of top 20% degree nodes across rest and IGT states on the given 20% threshold, respectively. Results of the permutation test revealed that there was a significant shift in key nodes between rest and IGT networks (*p* < 0.0002). Examination of the degree consistency images revealed where in the brain these important shifts occurred. For example, high consistency in the DMN was present across both images, particularly in the ventromedial prefrontal cortex. However, there was considerably higher consistency in the posterior component of the DMN (Precuneus/PCC) during rest compared to IGT. In addition, the network hubs that were consistently located in the visual cortex during rest were no longer consistent during the IGT. Examination of the IGT images revealed that there was an increase in the hub consistency among areas of the FPN from rest to IGT, particularly in the SPC and VLPFC. In addition, there was a significant increase in hub consistency in the superior medial prefrontal cortex and dorsal ACC from rest to IGT.

### FPN and DMN Comparison

**Figure 5:**
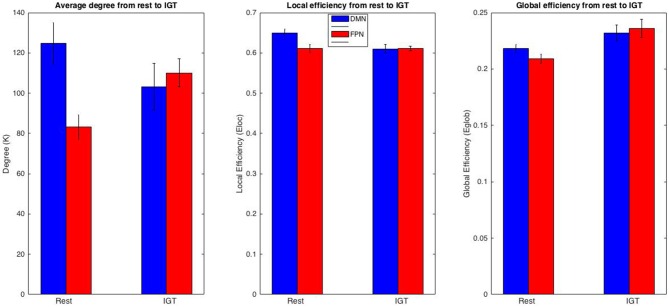
**FPN and DMN comparison.** Comparison of *K, E_loc_*, and *E*_glob_ between the DMN and FPN across rest and IGT. Metric values for the DMN are displayed in red and the FPN in blue. The DMN and FPN display differing patterns of change for *K* and *E*_loc_. In particular, a significant increase in *K* in the FPN and a significant decrease in *E*_loc_ in the DMN from rest to IGT. In addition, the differences in DMN and FPN in *K*, *E*_loc_, and *E*_glob_ during rest drop to non-significance during the IGT.

To examine regional changes in functional organization in the DMN and FPN between rest and the IGT, a repeated measures 2 (task) × 4 (session) ANOVAs were conducted for degree (*K*), local efficiency (*E*_loc_), and global efficiency (*E*_glob_; Figure 5) using data from each region. For all tests, there was no significant effect of session, or an interaction between task and session. For the DMN, there was a non-significant decrease in *K* from rest (*M* ± *SE =* 124.7 ± 10.317) to IGT (*M* ± *SE =* 103.21 ± 11.576) *F*_(1,8)_ = 4.529, *p* = 0.066, *η*^2^ = 0.361; *E*_loc_ significantly decreased from rest (*M* ± *SE* = 0.650 ± 0.009) to IGT (*M* ± *SE* = 0.61 ± 0.011), *F*_(1,8)_ = 40.452, *p* < 0.001, *η*^2^ = 0.835; and there was a non significant increase in *E*_glob_ from rest (*M* ± *SE* = 0.218 ± 0.004) to IGT (*M* ± *SE* = 0.232 ± 0.007), *F*_(1,8)_ = 4.224, *p* = 0.074, *η*^2^ = 0.346.

Consistent with our hypotheses of increased functional significance of FPN from rest to the IGT, *K* significantly increased from rest (*M* ± *SE =* 83.139 ± 6.185) to IGT (*M* ± *SE =* 110.058 ± 6.935) *F*_(1,8)_ = 19.019, *p* = 0.002, *η*^2^ = 0.704; *E*_loc_ did not show a significant difference between rest (*M* ± *SE* = 0.611 ± 0.01) to IGT (*M* ± *SE* = 0.611 ± 0.006) *F*_(1,8)_ = 0.001, *p* = 0.975, *η*^2^ < 0.001; and *E*_glob_ significantly increased from rest (*M* ± *SE* = 0.209 ± 0.004) to IGT (*M* ± *SE* = 0.236 ± 0.008) *F*_(1,8)_ = 12.198, *p* = 0.008, *η*^2^ = 0.604. In order to determine whether these changes were representative of all ROIs within the DMN or FPN, all of the above analyses were repeated for each individual ROI. All results revealed significant or non significant changes within each ROI in the same direction as the overall network (see Supplementary Tables A,B).

To test differences in network organization between the two networks, FPN and DMN, a repeated measures 2 (network) × 4 (session) ANOVA was conducted to test for any significant differences in network metrics (*K, E_glob_, E_loc_*) between the FPN and DMN during rest and the IGT. For all tests, there was no effect of session, or an interaction between sub-network and session. During rest, *K* was significantly higher in the DMN compared to the FPN, *F*_(1,8)_ = 11.091, *p* = 0.01, *η*^2^ = 0.581. During the IGT, there was no significant difference in *K* between the DMN and FPN, *F*_(1,8)_ = 0.172, *p* = 0.689, *η*^2^ = 0.021. During rest, *E*_loc_ was significantly higher in the DMN compared to the FPN, *F =* 38.951, *p* < 0.001, *η*^2^ = 0.830. During the IGT, there was no significant difference in *E*_loc_ between the FPN and DMN, *F* = 0.016, *p* < 0.902, *η*^2^ = 0.002. During rest, *E*_glob_ was significantly higher in the DMN compared to the FPN, *F*_(1,8)_ = 17.521, *p* = 0.003, *η*^2^ = 0.687. During the IGT, there was no significant difference in *E*_glob_ between the FPN and the DMN, *F*_(1,8)_ = 0.245, *p* = 0.634, *η*^2^ = 0.03.

### Correlations With IGT Performance

In order to determine the functional significance of network organization in the DMN and FPN during the IGT, a linear mixed-model was used to predict IGT performance from the observed network metrics of the two networks (Table 1). The results revealed that no metrics in the FPN or DMN were found to be significant predictors of IGT performance.

**Table 1:** **Results of linear mixed-model**.

Network	B	SE	Sig.
**DMN**
*K*	0.056	0.066	0.408
*E*_loc_	−70.352	99.743	0.489
*E*_glob_	152.077	144.697	0.304
**FPN**
*K*	0.078	0.102	0.453
*E*_loc_	−266.822	151.174	0.091
*E*_glob_	−82.3	142.146	0.569

*Estimated parameters (B) and standard errors (SE) from linear-mixed model predicting IGT performance from network metrics of the DMN and FPN. The results revealed that no metrics in the DMN or FPN were significantly associated with IGT performance*.

## Discussion

The first goal of this study was to examine global changes in network organization between rest and a complex decision-making task (i.e., IGT), and across sessions of the same task. There were significant changes in global network organization between rest and the IGT, as measured by *E_loc_* and *E*_glob_. Consistent with hypotheses, network organization, on average, shifted from a more clustered, locally-ordered topology, with efficient local information transfer during rest, to a more diffuse, globally-ordered topology, with efficient information transfer across the entire network during the IGT. However, as noted above, the statistical significance of the decrease in local efficiency from rest to IGT was not found to be entirely independent of threshold effects, so the local efficiency results should be interpreted with some caution. Given the complexity of the IGT, and the large number of coordinated processes needed to perform the task successfully, a shift from a more modular organization to a more integrated organization would be expected. A similar shift to a more globally integrated organization was observed from rest to a working-memory task (Rzucidlo et al., 2013). Thus, the shift from a local to distributed organization may be representative of a variety of task states.

In agreement with findings from other studies (Fransson, 2006; Harrison et al., 2008; Buckner et al., 2009; Rzucidlo et al., 2013), network organization stayed consistent in certain respects, especially for network metrics (at the whole-brain and region level) across sessions of the same task. Surprisingly, consistency was even found across sessions on the IGT, despite the significant learning component of the task. However, this study did not find significant differences in behavioral performance across four sessions of the IGT, inconsistent with previous studies (Bechara et al., 1994; Ernst et al., 2002; Lawrence et al., 2009). In fact, net positive advantageous selections were seen across all sessions, suggesting that participants on average were able to develop advantageous strategies in the first session. The number of selections in each block was large (*n* = 60), giving participants ample time to learn (unconsciously or consciously) at least some reward contingencies before the end of the first block. In fact, one study suggests that 30 card selections may be enough trials for participants to develop some kind of understanding of the reward contingencies associated with each deck (Maia and McClelland, 2004).

In addition, regional changes were examined in two prominent resting state networks, the FPN and the DMN. Examination of the spatial consistency of the top 20% degree nodes revealed important shifts in degree hubs from rest to IGT, contrary to some previous studies of network changes from rest to task states (Harrison et al., 2008; Buckner et al., 2009). Examination of network metric changes across rest and the IGT revealed important functional changes in network organization between the two states. For example, the DMN decreased in average degree from rest to IGT, while the FPN increased in average degree from rest to IGT. In addition, local efficiency decreased from rest to IGT in the DMN, and global efficiency increased from rest to IGT in the FPN. These results seem to suggest an increased functional role of the FPN in response to the task demands associated with the IGT, with a corresponding decrease in the overall and local connectivity of the DMN. Interestingly, a comparison of network metrics between the two networks revealed a functional dominance of DMN compared to the FPN during rest, but no such dominance during the IGT. This is consistent with previous findings (Cole et al., 2010, 2013) that the FPN is an especially important hub network in the integration of important information from all other networks. These findings suggest that the FPN may act as a flexible hub of the brain, integrating information from various networks, particularly during task states.

Together, these results suggest that the network organization of the brain changes in important ways in response to task demands. In terms of overall network organization, the brain shifts to a more globally, distributed processing network in response to the task demands of the IGT. This change co-occurs with, and may be facilitated by a shift in hub structure from DMN areas to FPN areas. In addition, the examination of the relative differences in network metrics between the DMN and FPN during rest and the IGT revealed that the increased cognitive demands of the IGT were associated with an increase and decrease in efficiency and connectivity in the FPN and the DMN, respectively. Thus, these results suggest that network organization changes in the FPN may be of central importance in the cognitive processes associated with the IGT. However, no significant association was observed between any of the network metrics in the DMN and FPN during the IGT, and net advantageous selections in the IGT. We believe the size of the sample (*n* = 9) may be the reason for the non-significant associations between the metrics of the FPN and DMN, but future studies are needed for any further conclusions.

## Conclusion

The primary goal of this study was to apply network-based analysis to the study of task-related changes in network organization, particularly the study of a complex decision-making task, the IGT. The results of this study suggest that network topology, in agreement with more recent research on task-related changes in network organization, is significantly changed between various cognitive states. This was found to be the case not only at the whole-brain level, but in particular sub-networks of interest, specifically the FPN and the DMN. In addition, hub nodes were found to shift across the two states, with greater average degree in the DMN compared to the FPN during the resting state, but no difference in degree between the two networks during the IGT. Local and global efficiency followed the same trend, confirming the FPN’s role as a flexible network hub coordinating processes across networks during task states.

The results of this study are obviously limited by the small sample size (*n* = 9). However, the repeated measures design of this study (4 sessions for each task) allows for unique insight into the network stability/changes between and within states. The results of this study suggest that the network analysis of repeated-measure fMRI designs may be fruitfully applied in other task settings. It is hoped that the present study encourages more research on task-related changes in functional network organization and demonstrates the utility of network-based analyses of functional neuroimaging data.

## Author Contributions

TB (Primary Author): created and conducted experiment, analyzed results, wrote the manuscript. PL: oversaw research, guidance over analyses, worked on manuscript. RL: guided and conducted analysis of results. AM: helped create, conduct and guided the implementation of the experiment and helped with preprocessing of data. DD: originator of the research project, oversaw research, guided analyses, worked on manuscript.

## Conflict of Interest Statement

The authors declare that the research was conducted in the absence of any commercial or financial relationships that could be construed as a potential conflict of interest.
