# Supplementary material for: Graph-Theoretical Study of Functional Changes Associated with the Iowa Gambling Task
Source: Front Hum Neurosci. 2016 Jun 27;10:314. doi: 10.3389/fnhum.2016.00314 (PMC4921456; doi:10.3389/fnhum.2016.00314)
Supplement: Supplementary file 1 [file DataSheet_1.pdf]

Supplementary Tables

Region	*Task*	*K*	*Eloc*	*Eglob*
VLPFC	Rest	**54.70 ± 5.26**	.585 ± .012	**.198 ± .004**
	IGT	**78.57 ± 6.62**	.583 ± .013	**.225 ± .007**
SPC	Rest	**112.06 ± 12.52**	.654 ± .008	**.219 ± .005**
	IGT	**150.88 ± 17.11**	.644 ± .008	**.244 ± .008**
DLPFC	Rest	**87.34 ± 11.88**	.610 ± .016	**.212 ± .005**
	IGT	**108..01 ± 9.05**	.621 ± .009	**.240 ± .007**
Dorsal ACC	Rest	**65.85 ± 9.75**	.575 ± .011	**.207 ±. 006**
	IGT	**90.20 ± 9.61**	.588 ± .011	**.235 ±. 009**

Supplementary Table A. **Metric Values for FPN regions across Rest and IGT.** (Ventrolateral prefrontal cortex: VLPFC, superior parietal cortex: SPC, dorsolateral prefrontal cortex: DLPFC, and dorsal anterior cingulate cortex: Dorsal ACC). Comparison of K, E_{loc}, and E_{glob} between rest and IGT for FPN ROIs. Metrics values that were found to be significantly different across rest and IGT ($p < .05$, uncorrected for multiple comparisons), as indicated by a 2 (task) × 4 (session) RM ANOVA, are listed in bold.

Region	*Task*	*K*	*Eloc*	*Eglob*
vmPFC	Rest	94.91 ± 12.69	**.626 ± .014**	.212 ± .004
	IGT	83.28 ± 11.19	**.582 ± .015**	.229 ± .009
Precuneus/PCC	Rest	**78.42 ± 5.25**	**.563 ± .009**	.191 ± .004
	IGT	**64.46 ± 4.01**	**.489 ± .010**	.207 ± .008
IPC	Rest	**118.01 ± 11.62**	**.628 ± .012**	**.215 ± .004**
	IGT	**100.57 ± 8.84**	**.605 ± .009**	**.231 ± .008**

Supplementary Table B. **Metric Values for DMN regions across Rest and IGT.** (ventromedial prefrontal cortex: vmPFC, precuneus/posterior cingulate cortex: Precuneus/PCC, inferior parietal cortex: IPC). Comparison of K, E_{loc}, and E_{glob} between rest and IGT for DMN ROIs. Metrics values that were found to be significantly different across rest and IGT ($p < .05$, uncorrected for multiple comparisons), as indicated by a 2 (task) × 4 (session) RM ANOVA, are listed in bold.
